# Encouraging medical students to become surgeons? Impact of psychological and surgical factors on career choice at medical school

**DOI:** 10.3205/zma001676

**Published:** 2024-04-15

**Authors:** Sandra Brügge, Veronika Günther, Ingolf Cascorbi, Nicolai Maass, Zino Ruchay, Martin R. Fischer, Johanna Huber, Ibrahim Alkatout

**Affiliations:** 1 University Hospitals Schleswig-Holstein, Campus Kiel, Clinic for Obstetrics and Gynecology, Kiel, Germany; 2 University Hospitals Schleswig-Holstein, Campus Kiel, Institute for Experimental and Clinical Pharmacology, Kiel, Germany; 3 Ludwig-Maximilians University Munich, Institute for Didactics and Training Research in Medicine, Munich, Germany

**Keywords:** post-graduate medical training, interest in surgery, self-efficacy expectations, resilience, stress

## Abstract

**Aim:**

Training decisions are viewed as a problem by the majority of medical students.

In the present study we compared sociodemographic and psychological characteristics of students who are interested in surgical training to those who preferred a non-surgical specialty. Furthermore, we examined whether students who wish to be trained as surgeons performed better than their non-surgical counterparts in a course designed to acquire skills in minimally invasive surgery.

**Method:**

From October 2020 to January 2021 we performed a cross-sectional survey among 116 medical students prior to their year of practical training at Christian-Albrechts University in Kiel. Based on their intended field of specialization, the students were divided into a non-surgical and a surgical group. Sociodemographic and psychological characteristics such as self-efficacy expectations, resilience and stress perception were evaluated and compared between groups. Simultaneously, we compared their surgical performance in two laparoscopic exercises and their self-assessment as surgeons. Statistical differences between the training groups were determined by the Mann-Whitney U test or Pearson’s Chi square test.

**Results:**

Ninety-two students participated in the study, of whom 64.1% intended to train in a non-surgical specialty and 35.9% in a surgical specialty. Students who wished to be trained as surgeons had higher general self-efficacy expectations (p<0.001) and greater resilience (p=0.009). However, on comparison they had a lower stress level (p=0.047). The inter-group comparison of training results and self-assessment as surgeons revealed no unequivocal differences in surgical performance.

**Conclusion:**

Interest in surgical specialties is correlated, among other factors, with the strength of psychological skills such as general self-efficacy expectations, resilience and stress perception. Early attention to these psychological resources in academic training might assist medical students in future career choices.

## Zusammenfassung

**Zielsetzung:**

Die Weiterbildungsentscheidung wird von dem Großteil der Medizinstudierenden als schwierig empfunden.

Die vorliegende Studie vergleicht die soziodemographischen und psychologischen Merkmale von Studierenden, die sich für ein operatives Weiterbildungsfach interessieren, mit denen, die ein nicht-operatives Fach bevorzugen. Außerdem wird untersucht, ob die Studierenden mit einem operativen Weiterbildungswunsch in einem Kurs zum Erlernen minimal-invasiver Fertigkeiten besser abschneiden als die nicht-operative Vergleichsgruppe.

**Methodik:**

Im Zeitraum von Oktober 2020 bis Januar 2021 wurde eine Querschnittsbefragung von 116 Medizinstudierenden vor dem Praktischen Jahr an der Christian-Albrechts-Universität zu Kiel durchgeführt. Anhand ihres geplanten Weiterbildungsfaches wurden die Studierenden zwei Gruppen („nicht-operativ“ und „operativ“) zugeordnet und ihre soziodemographischen und psychologischen Merkmale wie allgemeine Selbstwirksamkeitserwartung, Resilienz und Stresswahrnehmung zwischen den Gruppen evaluiert. Analog erfolgte ein Vergleich der operativen Leistung in zwei laparoskopischen Operationsübungen sowie der operativen Selbsteinschätzung. Statistische Unterschiede zwischen den verglichenen Weiterbildungsgruppen wurden mittels Mann-Whitney-U-Test oder Chi-Quadrat-Test nach Pearson ermittelt.

**Ergebnisse:**

Insgesamt nahmen 92 Studierende an der Untersuchung teil, davon planten 64,1% ihre ärztliche Weiterbildung in einem nicht-operativen und 35,9% in einem operativen Fach. Die Studierenden mit einem operativen Weiterbildungswunsch zeigten eine höhere allgemeine Selbstwirksamkeitserwartung (p<0,001) und eine höhere Ausprägung der Resilienz (p=0,009). Außerdem wiesen sie ein im Vergleich niedrigeres Stresslevel (p=0,047) auf. Der Vergleich der Trainingsergebnisse und operativen Selbsteinschätzung beider Gruppen zeigte keine eindeutigen Unterschiede für die operative Leistung.

**Schlussfolgerung:**

Das Interesse für ein operatives Fachgebiet korreliert unter anderem mit der Ausprägung von psychologischen Fähigkeiten wie der allgemeinen Selbstwirksamkeitserwartung, Resilienz und Stresswahrnehmung. Eine frühzeitige Berücksichtigung dieser Ressourcen in der akademischen Ausbildung könnte Medizinstudierende auf ihrem beruflichen Entscheidungsweg unterstützen.

## Introduction

The average span of a doctor’s professional life is 40 years. A small percentage of medical students (about 7%) will have established their future specialty of choice at the start of medical school [1]. The large majority of medical students make their career decisions during, and most frequently at the end, of medical school. In this regard, a number of factors have been investigated over several generations and identified as factors influencing the decisions. A systematic review identified five main categories of associated factors:

1. Structure of the medical curriculum,
2. Characteristics of students such as age and personality,
3. Values and interests of students,
4. Professional requirements and
5. Personal experiences in medical specialties [2].

Especially the factors based on professional and career requirements, such as workload or the anticipated income, and work-life balance appear to be of increasing importance in career decisions [2]. However, the presentation of a medical specialty by role models at medical school, in curricular courses or clerkships, is an important factor and plays a significant roles in making career decisions [3].

Personal and psychological characteristics of students and graduates have been scarcely investigated in connection with medical training decisions. 

The psychological construct of self-efficacy expectations, as postulated by Bandura (1977), forms the core of professional goals and success [4], [5]. Independent of medicine, an association between professional careers and psychological resources, especially resilience, has been observed in all occupational fields. High resilience is associated with work satisfaction and increased performance at work [6], [7]. The stress levels experienced by medical students also play an important role because these are higher than those experienced by students in other branches of study and are associated with psychiatric illnesses such as depression [8].

Knowledge and comprehension of these decisive factors is of great interest because all surgical specialties are confronted with enormous recruitment difficulties [9]. Ensuring the existence and diversity of all specialties dealing with patient care in a sustained manner through several generations is a major challenge [10]. The planned reform of medical license regulations (ÄApprO), in accordance with the national competency-based learning objectives for medicine or NKLM [https://nklm.de/zend/menu], is now more strongly oriented towards future role models for doctors, but offers no support in making career decisions as a specific curricular learning objective. 

In the present study we investigated associations between the psychological resources of resilience, self-efficacy expectations, individual stress perception, and future career decisions of medical students. Knowledge of these associations would permit early assistance and accompaniment of medical students in their professional career choices. The following issues will be addressed:

1. Is the decision in favor of a surgical specialty correlated: a) with higher general self-efficacy expectations, b) with greater resilience, c) negatively with stress perception?
2. Does an association exist between surgical performance (self-assessment and objective performance in a training program for acquiring skills in minimally invasive surgery) and medical career decisions in favor of surgery during medical school?

## Method

**Figure 1:**
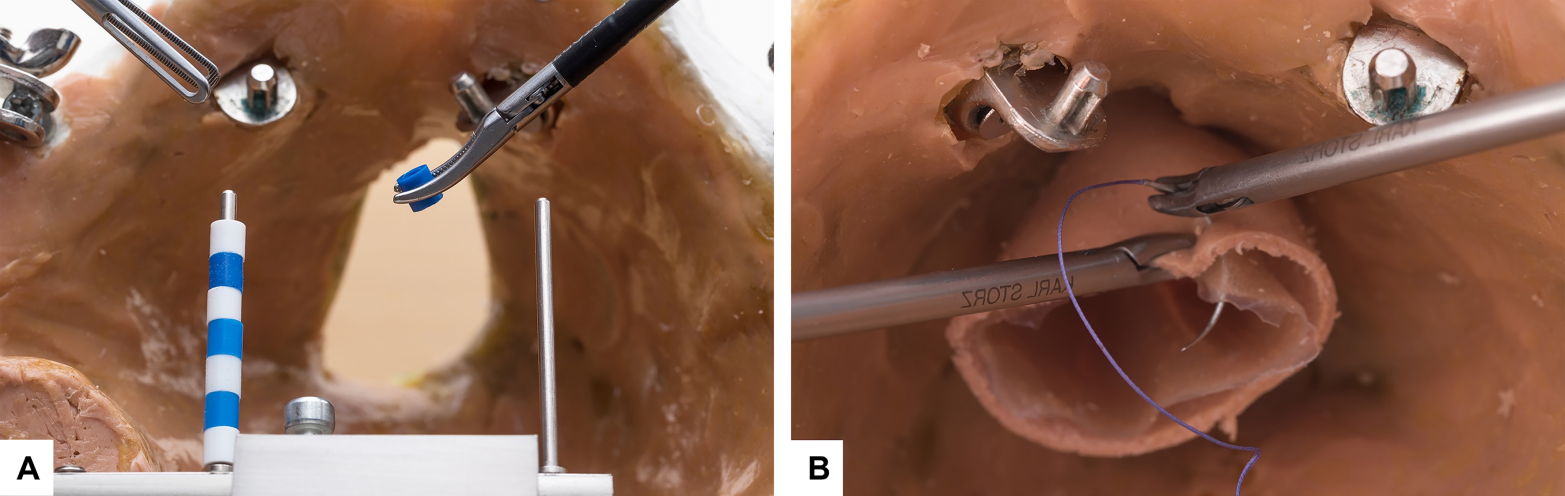
Surgical exercises The two surgical exercises performed in the course for acquiring skills in minimally invasive surgery. (A) Coordination exercise with pearls (B) Knotting exercise

We performed a cross-sectional survey among medical students in their fifth year of medical school at Christian-Albrechts University in Kiel. The survey was conducted during the gynecological clerkship at the Clinic of Obstetrics and Gynecology, University Clinic of Schleswig-Holstein, Campus Kiel. In this phase of medical school, the large majority of the students focus on planning their upcoming year of practical internship and thus on their future medical career decisions [1]. The gynecological clerkship is focused on training in surgery at medical school. This includes a course in minimally invasive surgery which provides the attendees an opportunity to gain personal experience in surgery as well as acquire practical skills while at medical school. The survey was conducted from October 2020 to January 2021 with the aid of the web-based survey software evasys, Version 8.1 (evasys GmbH, Lüneburg, Germany). In addition to general demographic data, the survey inquired about the students’ future field of specialization based on the specialties listed in the training policy for medical specialties issued by the Medical Chamber of Schleswig-Holstein [11]. The selected specialties were divided into two categories for purposes of statistical evaluation: surgical and non-surgical. Surgical abilities were registered as objective performance (the outcome of courses for acquiring surgical skills) and subjective performance (individual self-assessment of course results), and were assigned to the surveyed persons in pseudonymized form. The results refer to a practical training course focused on the acquisition of basic skills in minimally invasive surgery during the gynecological clerkship. The practical exercises were performed in a conventional laparoscopic environment on a pelvitrainer (Realsimulator 2.0, based on a female anatomy model, from the Pelvic School of Saarbrücken – Endodevelop) and instruments of Karl Storz Company (KARL STORZ GmbH & Co. KG, Tuttlingen). Two established exercises were used for the investigation (see figure 1 (Fig. 1)) [12], [13]. The coordination exercise with pearls is a basic exercise for which the students had to transfer eight pearls from a left-sided to a right-sided rod, followed by a motion of the pearls in the opposite direction. The total number of shifted pearls and the time taken to complete the exercise were registered. The mean period of time for moving one pearl (in seconds) was calculated from the result. The “knotting exercise”, on the other hand, simulates a complex step in gynecological surgery: closure of the vaginal vault after total laparoscopic hysterectomy. It is more difficult than the pearls exercise. The knotting exercise consists of two individual sutures placed bilaterally consisting of three superimposed knots (an initial double knot followed by two single knots, one of which had to be knotted in the opposite direction). The total time taken for both knots (in seconds) was used for the evaluation.

The students’ *self-assessment* of their surgical performance was registered on a three-point categorial scale on which the students could rate their own results into one of three categories:

1. above average,
2. average, or
3. below-average.

The psychological characteristics listed below were determined from validated questionnaires. For the registration of general self-efficacy expectations we used the 10-item short Scale for General Self-Efficacy Expectation (SWE) [4], [14]. The items had to be rated on a four-point Likert scale (1=absolutely inappropriate, 2=inappropriate, 3=appropriate, 4=absolutely appropriate. The individual values were derived by adding the individual item scores, which yielded an overall score between 10 (low self-efficacy expectations) and 40 (high self-efficacy expectations). The students’ *resilience* status was registered on the 10-item Connor-Davidson Resilience Scale (CD-RISC-10) [6], [15]. The questionnaire measures resilience on the basis of 10 items, on a five-point Likert scale from 0 (absolutely false) to 4 (nearly always true). The answers reflect the students’ frame of mind during the last four weeks. The total score was derived from the sum of the individual item scores. Scores could range from minimum 0 (low resilience) to 40 (high resilience). Subjective stress perception was registered on the 10-item *Perceived Stress Scale* (PSS-10) [16], [17]. The responses referred to the last four weeks and the questions were answered on a five-point Likert scale (0=never, 1=almost never, 2=sometimes, 3=quite often and 4=very often). The values were added to determine the total score; the items 4, 5, 7 and 8 (concerning self-efficacy) were measured by reversed polarity. The total score could range from 0 to 40 points. The latter is associated with the highest stress level.

### Statistical analysis 

Microsoft Office Excel^®^ 2007 (Microsoft Corporation, Redmont, WA, USA) and IBM SPSS Statistics 28 (SPSS Inc. an IBM Company, Chicago, IL) were used for statistical analysis. Quantitative variables were presented descriptively as medians and interquartile ranges (IQR). The statistical analysis was based on the available data. Missing values were not replaced. A student’s choice of specialty was determined as the dependent variable (surgical vs. non-surgical).

Sociodemographic data such as gender and age, psychological characteristics such as general self-efficacy expectations, resilience, and stress perception, and surgical performance were viewed as independent variables in respect of their impact on the selected target variable. The assignment of students to one of the two groups depended on their decision to opt for a surgical or non-surgical specialty. Differences between the two groups in regard of quantitative variables were tested with the Mann-Whitney U test. In addition, differences were evaluated with the measure of effect size according to Cohen’s d (d). Comparisons between groups in regard of qualitative variables, gender and self-assessment of surgical skills was performed by Pearson’s Chi square test. The level of significance was set to 5% and two-tailed tests were used. Alpha adjustment for multiple testing was not performed. Hence the results have an exploratory and descriptive character, and were interpreted accordingly. 

## Results

Of 116 surveyed students, 92 completed the survey. An overview of sociodemographic data is provided in table 1 (Tab. 1). The mean number of semesters at the time of the investigation was 10 and the mean age of the students was 25 years. Fifty-nine students (64.1%) planned to train in a non-surgical specialty after their board examination and 33 students (35.9%) planned to train in a surgical specialty. Gender did not differ significantly between the two training groups (p=0.305) (see table 2 (Tab. 2)). Age also did not differ significantly: 25 years (IQR 24.0-27.5) in the non-surgical group and 25 years (IQR 24.0-29.0) in the surgical group (p=0.815). The classification of specialties and the frequency of the individual specialties are summarized in attachment 1 . 

**Figure 2:**
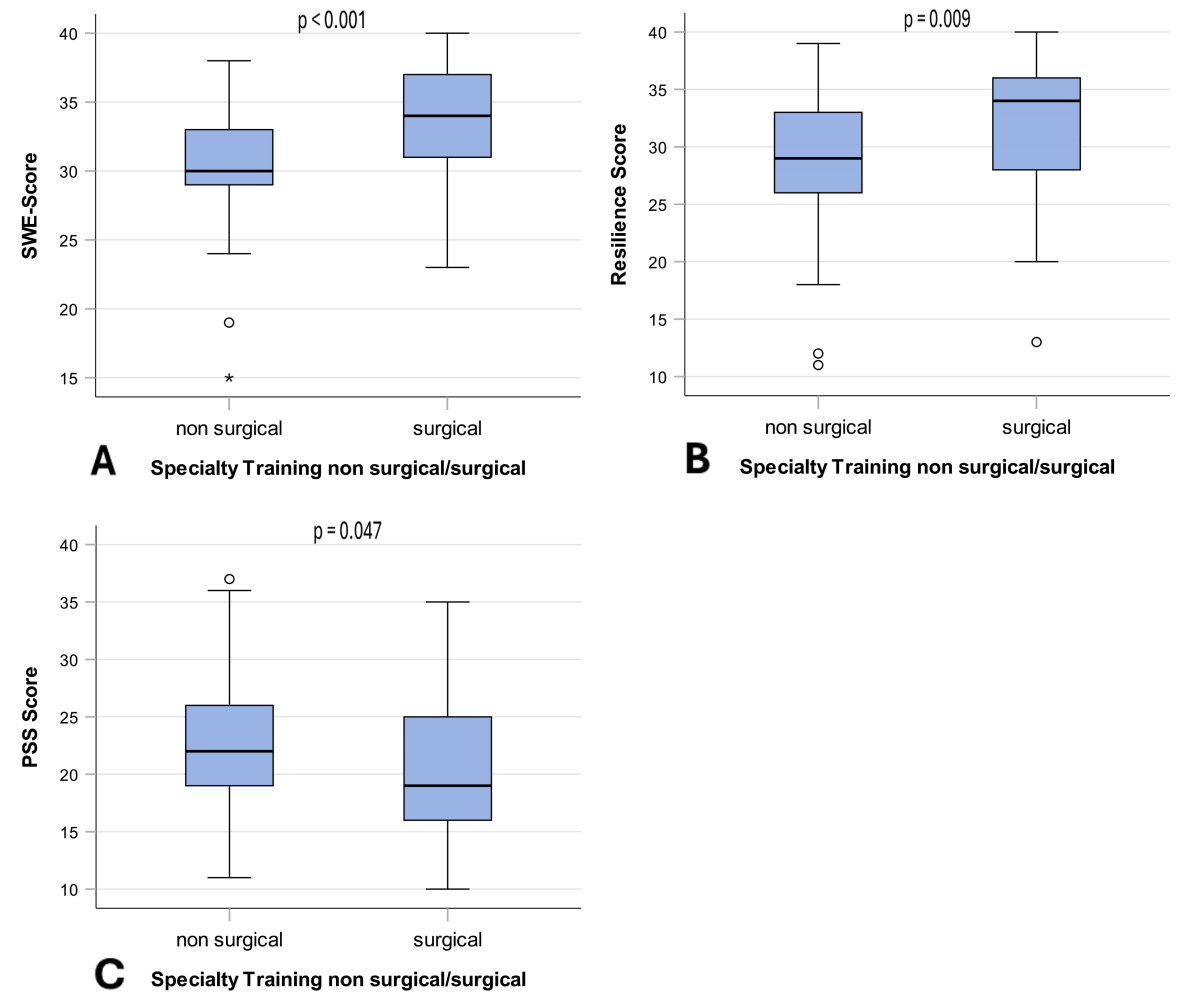
Psychological characteristics in the career choice groups The figure shows the respective scores for the psychological characteristics of (A) Self-efficacy expectations (SWE), (B) Resilience and (C) Stress perception (PSS) in boxplot diagrams, and compares the two career choice groups (non-surgical and surgical) (n=92). Each diagram shows medians, 1^st^ and 3^rd^ quartiles with the interquartile range (IQR) and the error bar (IQR * 1.5). Differences between groups were tested with the Mann-Whitney U test. The significance level was set to 5% and two-tailed tests were performed. Lower PSS scores were associated with a lower subjective perception of stress.

Students who wished to train in surgery had significantly higher scores of general self-efficacy expectations (median 34, IQR 31-37 and median 30, IQR 29-33, p<0.001, d=0.757) and resilience (median=34, IQR 28-36, and median=29, IQR 28-36, p=0.009, d=0.498). Subjective stress levels were significantly lower among students not interested in a surgical specialty (median=19, IQR 16-25 and median=22, IQR=19-26, p=0.047, d=0.431) (see table 3 (Tab. 3) and see figure 2 (Fig. 2)). 

**Figure 3:**
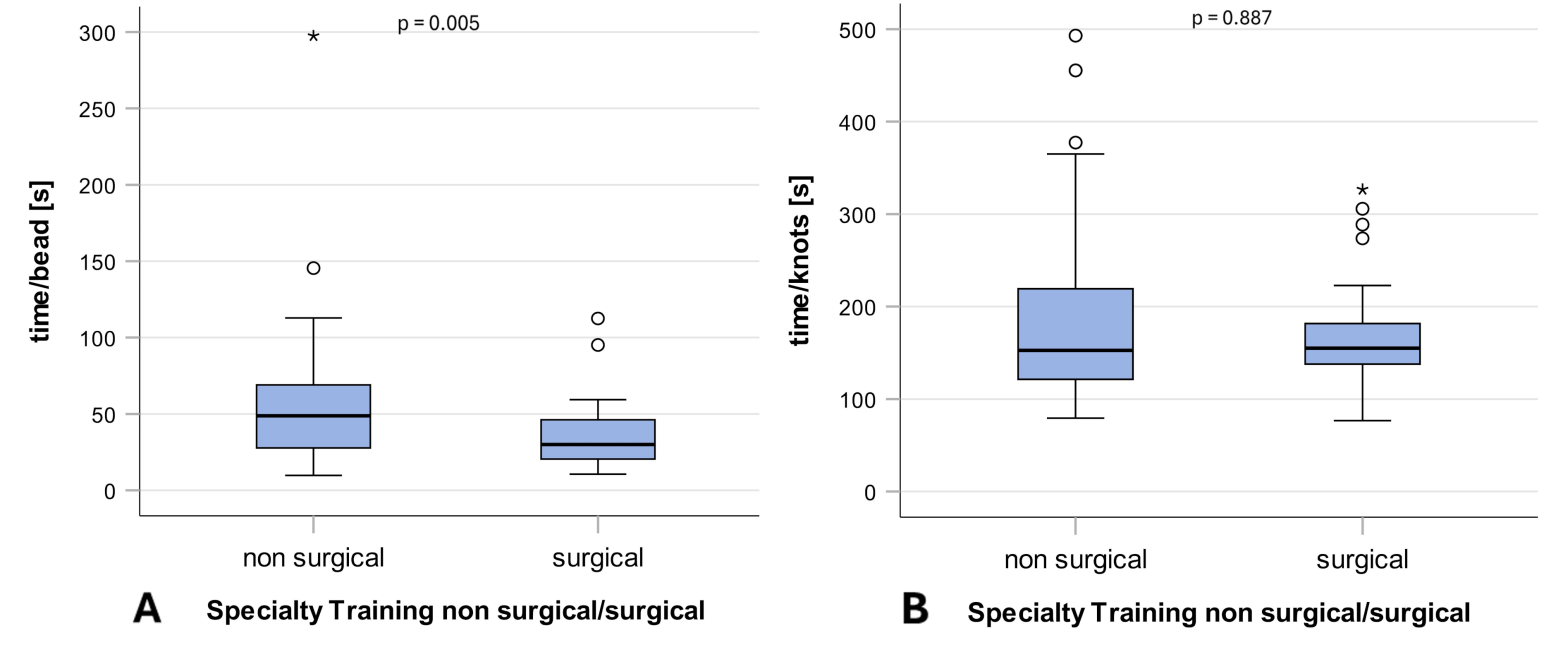
Performance in surgical exercises in the career choice groups The figure shows performance in the two surgical exercises: (A) Coordination exercise with pearls and (B) Knotting exercise, in boxplot diagrams, comparing the two career choice groups (non-surgical and surgical) (n=92). Each diagram shows medians, 1^st^ and 3^rd^ quartiles, the interquartile range (IQR), and the error bar (IQR * 1.5). Differences between the two groups were tested with the Mann-Whitney U test. The level of significance was set to 5% and two-tailed tests were performed.

In the surgery course for the acquisition of skills in minimally invasive surgery, students of the surgery group completed the coordination exercise with pearls significantly faster (median=30.0, IQR 20.4-47.3 and median=48.8, IQR 27.5-70.7, p=0.005, d=0.513). The knotting exercise revealed no significant difference between the two investigated groups (median=155.0, IQR 133.5-186.3, and median=152.7, IQR=121.0-230.0, p=0.887, d=0.181) (see table 4 (Tab. 4) and see figure 3 (Fig. 3)). 

Ninety students responded to the question about their self-assessment of results in the surgical exercises. The number of students who rated their course results as above-average was higher in the surgery group [34.4% (n=11) versus 25.7% (n=15)], although the difference did not achieve statistical significance (p=0.305). 62.1% (n=36) and 62.5% (n=20) of the students from the non-surgical and surgical group, respectively, gave their performance an average rating. With regard to self-assessment, fewer students of the surgical group gave their course grades a below-average rating: 3.1% (n=1) versus 12.1% (n=7) (see table 5 (Tab. 5)). 

## Discussion

In the present study we evaluated the impact of psychological and surgical factors on the career choices of 92 medical students. Knowledge of these factors will aid educators in devising specific decision-making tools for students. The existing published literature on the subject is limited [18], [19], [20].

In a survey of doctors, Hojat et al. found differences in empathy in the various specifications for medical specialists [21]. Psychiatrists had significantly higher empathy values than doctors working in a surgical specialty (e.g. general surgery, neurosurgery, obstetrics and gynecology). Possibly, persons with different degrees of interpersonal skills are more likely interested in specific specialties and this may have had an impact on their choice of specialty in the past as well [21]. Analogously, our investigation also revealed a correlation between psychological factors and interest in a medical specialty. In contrast to the above mentioned study, in our survey those interested in surgery had higher levels of self-efficacy and resilience.

### Impact of self-efficacy on career choices

General self-efficacy expectations are interpreted as the individual’s confidence in his/her options and competences in coping with different life situations, and is positively correlated with individual well-being [4], [22]. The well-being of doctors is also significant for the medical care of patients and is negatively associated with a poorer outcome of treatment [23]. In the present investigation, students who wished to train in surgery had higher self-efficacy expectations. To our knowledge, this is the first direct comparison of medical students in regard of their vocational preferences and self-efficacy. A cross-sectional survey of 202 medical students (all semesters) showed that general self-efficacy expectations are associated with preferences for a specific specialty [24]. In a cross-sectional survey, Heinen et al. compared the self-efficacy expectations of medical students (n=360) during their first year of medical school with those of active surgeons. Interestingly, students had significantly lower self-efficacy expectations [25]. The authors concluded that students are initially called upon to adjust to the specific challenges of medical school, and their self-efficacy expectations increase in the course of their medical education. A comparison with doctors working in conservative non-surgical specialties was not performed. 

A cross-sectional survey of general self-efficacy expectations was conducted in 2019 among assistant doctors in surgery [22]. The 179 doctors had higher self-efficacy expectations than students (of all courses) and managers. A positive correlation was also noted between general self-efficacy expectations and psychological well-being.

### Resilience of medical students 

The present investigation revealed higher values of resilience among students who planned to train in a surgical specialty. Two representative cross-sectional studies from Canada (2014) and the USA (2017) showed that medical students had a lower level of resilience than the normal population. In the American study, students in their third year of medical school were compared to those in their fourth year; the latter had higher resilience scores [26], [27]. In a large cross-sectional survey conducted in 2018, the resilience of 613 medical graduates (Bavarian Graduate Study Medicine) was registered and viewed in detail [6]. This survey was conducted about a year after the third medical examination. The mean scores of doctors were higher than those of medical students in the two previously mentioned studies (Canada and the USA). The direct comparison of our resilience scores with those in the above mentioned studies revealed that students who desired to train in a surgical specialty had the highest scores among students, but lower scores than doctors in their first year of training in all specialties [6]. The authors of the study concluded that the relatively high resilience scores of their respondents were attributable to their brief experience of medical work, and expected their resilience to improve and stand the test of time in their subsequent medical career [6].

### Impact of stress perception at medical school

Stress perception at medical school has been addressed in several studies [28], [29]. The present investigation revealed significant differences between students in regard of their scores on the Perceived Stress Scale. Students who wished to train in a surgical specialty had a lower level of stress than students with a non-surgical preference. The published literature reveals two interesting studies on the subject. A well-known US-American cross-sectional survey of 290 students showed higher stress levels among students of dentistry than those of human medicine [30]. In a German cross-sectional study conducted in 2014, the specific stress levels of 321 medical students in their first year at medical school were investigated. Compared to the general population and to medical students in their second year of medical school, the authors found significantly higher stress levels in the investigated group [25]. One explanation is that stress perceptions undergo dynamic changes during medical school (a higher stress level in the first year than in the second year, and a higher stress level compared to the general population) and is adjusted to the existing challenges. A higher stress level per se compared to other educational courses is probably related to specific stresses such as contact with suffering and dying patients [28]. 

### Impact of surgical performance on medical career choices 

As we have no suitable instrument to register surgical self-efficacy as a specific entity [22], the question of subjective self-assessment of surgical performance was addressed and interpreted in addition to general self-efficacy. A student’s personal assessment of his/her performance in surgical training did not differ significantly between the two groups. Nevertheless, students who wished to opt for surgery gave their surgical performance an above-average rating more frequently (34.4% vs. 25.7%). In contrast, students in the non-surgical group gave their performance a below-average rating more frequently (12.1% vs. 3.1%). In terms of objective performance, we found no association between the two groups. Our results permit no conclusions about the reasons for the students’ estimations. One explanation is provided by a large cross-sectional study from China comprising 1930 medical students [31], which revealed a significant association between higher self-efficacy expectations, intrinsic motivation, and academic success. Interestingly, male students rated their intrinsic motivation higher and had poorer academic results than female students.

Conversely, however, experiences in surgery (such as training and mentoring) also influence the vocational decisions, satisfaction, and performance of students and doctors [32], [33]. Early participation in a surgical convention or congress during medical school led to greater interest in a surgical specialty for 37.6% of the surveyed medical students (second pre-clinical year of study), while 60.3% reported no such effect [34]. In a cross-sectional study, 64 medical students in different phases of medical school underwent a minimally invasive training program; the impact of the course on their interest in surgery and choice of specialty was investigated [18]. 45.3% of the students said that the surgical training course had a positive impact on their career choice in favor of surgery. A significant increase in the choice of surgery as a specialty was not seen in a specific evaluation of students interested in surgery and those not interested in surgery. Furthermore, both groups achieved similar results in the non-invasive surgical exercises.

### Limitations of the present study

This was a single-center cross-sectional study with a specific sample size. The results and their significance cannot be generalized or applied to other populations. The existing data refer to the self-assessment of medical students during their fifth year of medical school and not their actual choice of future specialization. Longitudinal investigations beyond the third medical board examination should follow. We performed an isolated analysis of the variables needed to answer the research questions. We did not consider confounding variables such as personal or financial reasons for or against the choice of a specific specialty.

## Conclusions

The results of the present study show that the psychological characteristics of medical students have an impact on their future career choices and their future professional lives as doctors. Especially the choice between a surgical and a non-surgical specialty appears to be influenced by these factors. Although further studies and analyses, especially longitudinal studies and multiple regression analysis will be needed to provide final answers, the current data may be used to develop supportive tools for making career decisions. Early encouragement of, and emphasis on interests, and the reinforcement of psychological resources could assist students in their decision-making processes beyond medical school, and strengthen their resolve to pursue a certain career. Cognizance of these factors would be a first step towards overcoming the previously mentioned recruitment problems faced in the medical profession.

## Acknowledgements

The authors thank all students for their willing cooperation and wish them all the best for the future. Furthermore, the authors thank Saskia Struck for her management of the Kiel School of Gynaecological Endoscopy, Ulrike von Hehn of medistat.de Company for her statsitical analysis of the results, Julian Pape for his support in layout, and Karl Storz Company for their generous provision of the entire training units of minimally invasive surgery for all students.

The lead author is especially grateful to the management of the Master of Medical Education (MME) course at Heidelberg University for enabling and supporting the current study in terms of planning and evaluation.

## Notes

### Ethics

All participants consented to their participation in the study. The study was approved by the ethics committee of Christian Albrechts University of Kiel (D 448/21). 

### Authors’ ORCIDs

- Veronika Günther: [0000-0002-1132-1315]
- Ingolf Cascorbi: [0000-0002-2182-9534]
- Nicolai Maass: [0000-0002-1430-4676]
- Zino Ruchay: [0000-0001-5439-8817]
- Martin R. Fischer: [0000-0002-5299-5025]
- Johanna Huber: [0009-0005-9518-730X]
- Ibrahim Alkatout: [0000-0002-7194-6034]

## Competing interests

The authors declare that they have no competing interests. 

## Supplementary Material

Future specialties of choice and categories

## Figures and Tables

**Table 1:**
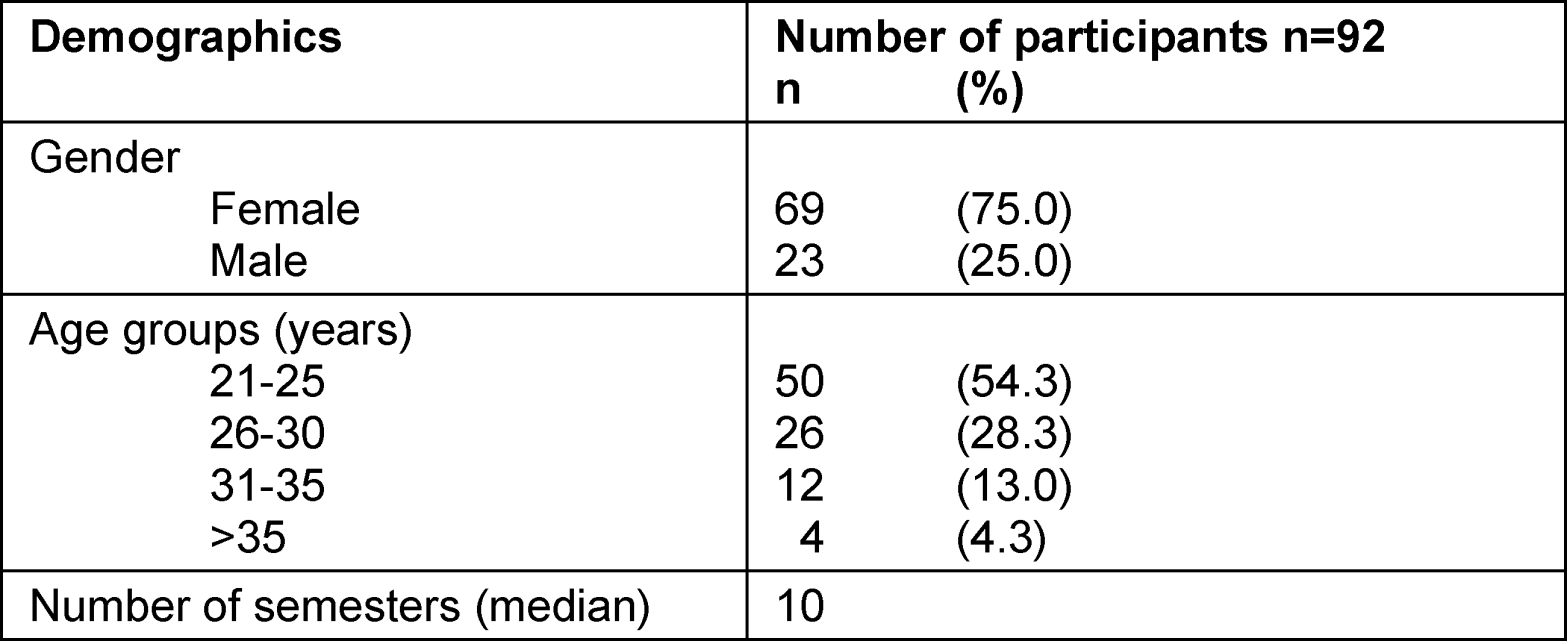
Sociodemographic characteristics

**Table 2:**
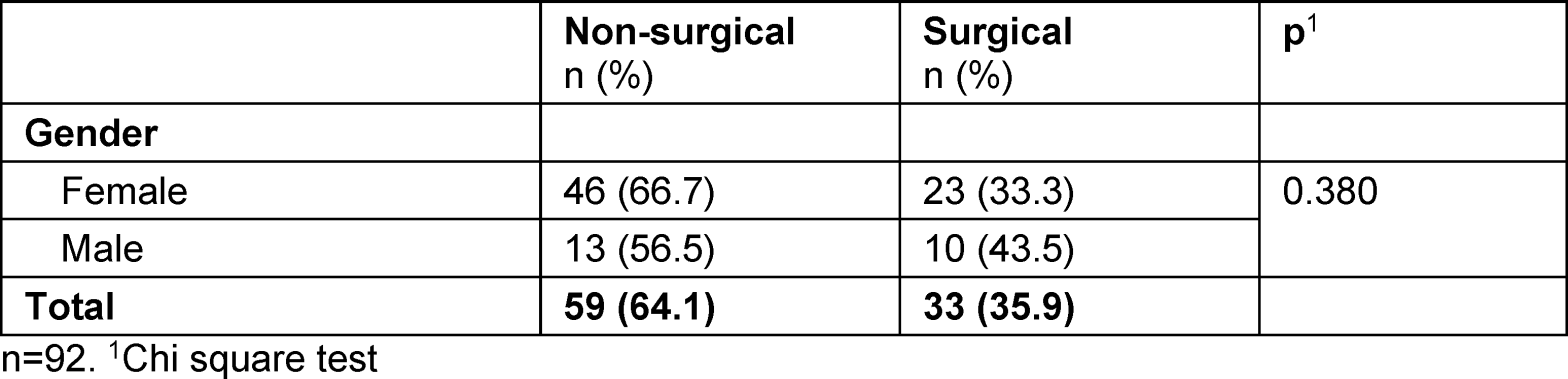
Gender-specific career choice

**Table 3:**
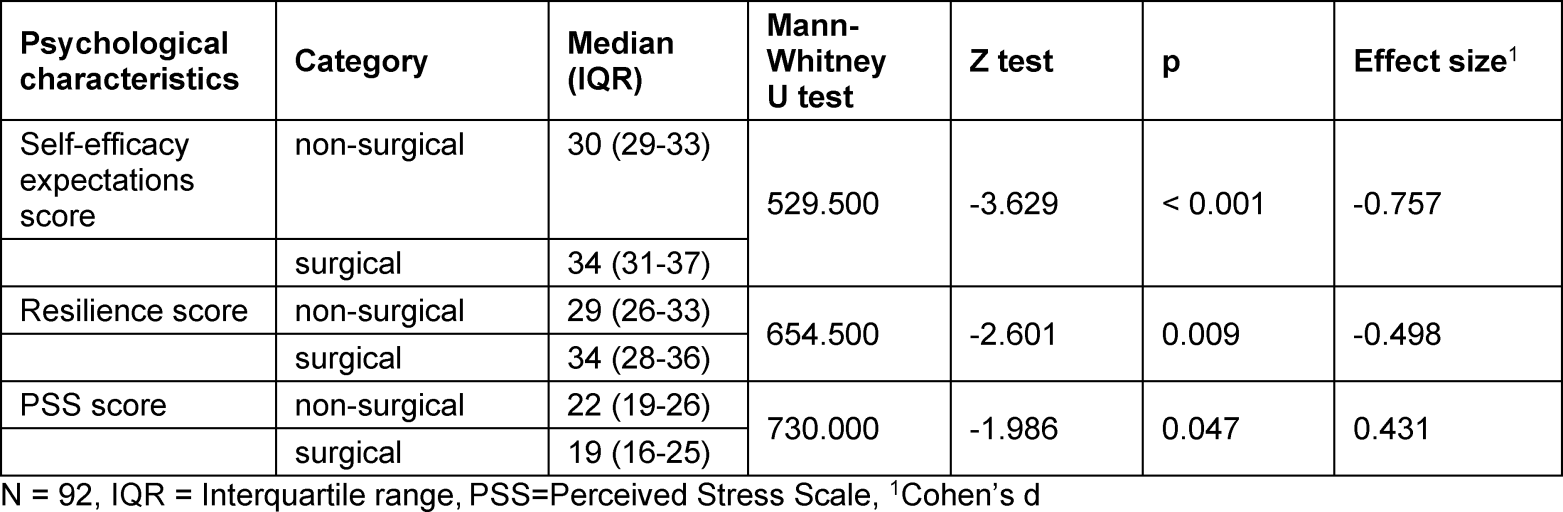
Psychological characteristics in the career choice groups

**Table 4:**
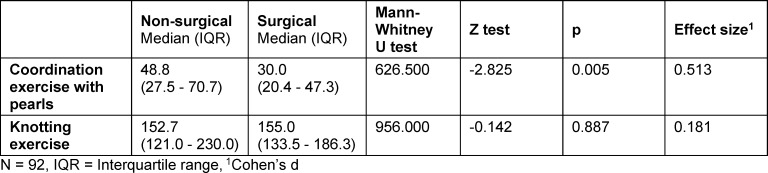
Surgical performance of the career choice groups

**Table 5:**
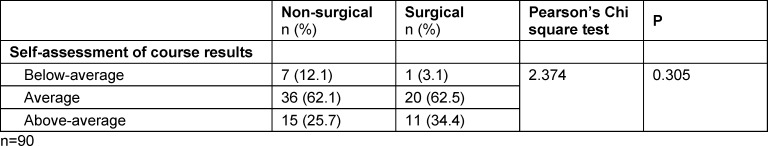
Self-assessment as surgeons of the career choice groups
