# Supplementary material for: Encouraging medical students to become surgeons? Impact of psychological and surgical factors on career choice at medical school
Source: GMS J Med Educ. 2024 Apr 15;41(2):Doc21. doi: 10.3205/zma001676 (PMC11106567; doi:10.3205/zma001676)
Supplement: Future specialties of choice and categories [file JME-41-21-s-001.pdf]

Attachment 1: Future specialties of choice and categories

Specialty	Frequency: n = 92		Category
	n	(%)	
Obstetrics and Gynecology	18	(19.6)	surgical
Internal medicine	13	(14.1)	non-surgical
Anesthesiology	10	(10.9)	non-surgical
Pediatric medicine	9	(9.8)	non-surgical
General medicine	7	(7.6)	non-surgical
General surgery	6	(6.5)	surgical
Orthopedics and Trauma Surgery	6	(6.5)	surgical
Psychiatry	4	(4.3)	non-surgical
Radiology	3	(3.3)	non-surgical
Urology	3	(3.3)	surgical
Neurosurgery	2	(2.2)	surgical
Pathology	2	(2.2)	non-surgical
Forensic medicine	2	(2.2)	non-surgical
Ophthalmology	1	(1.1)	non-surgical
Plastic surgery	1	(1.1)	surgical
Ear, Nose and Throat Medicine	1	(1.1)	surgical
Dermatology and Venerology	1	(1.1)	non-surgical
Neurology	1	(1.1)	non-surgical
Transfusion medicine	1	(1.1)	non-surgical
Others	1	(1.1)	non-surgical

Attachment 1 to Brügge S, Günther V, Cascorbi I, Maass N, Ruchay Z, Fischer MR, Huber J, Alkatout I. *Encouraging medical students to became surgeons?Impact of psychological and surgical factors on career choice at medical school.* GMS J Med Educ. 2024;41(2):Doc21. DOI: 10.3205/zma001676
